# Multi-Dimensional Enhanced Seizure Prediction Framework Based on Graph Convolutional Network

**DOI:** 10.3389/fninf.2021.605729

**Published:** 2021-08-19

**Authors:** Xin Chen, Yuanjie Zheng, Changxu Dong, Sutao Song

**Affiliations:** ^1^School of Information Science and Engineering at Shandong Normal University, Jinan, China; ^2^School of Computer Science and Engineering at Southeast University, Nanjing, China; ^3^Key Lab of Intelligent Computing and Information Security in Universities of Shandong, Shandong Normal University, Jinan, China; ^4^Shandong Provincial Key Laboratory for Novel Distributed Computer Software Technology, Shandong Normal University, Jinan, China; ^5^Institute of Biomedical Sciences, Shandong Normal University, Jinan, China

**Keywords:** epilepsy EEG signal, seizures prediction, multichannel relationship, graph convolutional network, space-time prediction

## Abstract

In terms of seizure prediction, how to fully mine relational data information among multiple channels of epileptic EEG? This is a scientific research subject worthy of further exploration. Recently, we propose a multi-dimensional enhanced seizure prediction framework, which mainly includes information reconstruction space, graph state encoder, and space-time predictor. It takes multi-channel spatial relationship as breakthrough point. At the same time, it reconstructs data unit from frequency band level, updates graph coding representation, and explores space-time relationship. Through experiments on CHB-MIT dataset, sensitivity of the model reaches 98.61%, which proves effectiveness of the proposed model.

## 1. Introduction

Epilepsy is a chronic disease caused by brain dysfunction, which is characterized by sudden and transient (Jia et al., 2004). In the study of seizure symptoms, EEG plays an important role. It is an important auxiliary technology for epilepsy diagnosis (Yuan et al., 2012). In traditional diagnosis of epilepsy symptoms, EEG data is often analyzed by experienced doctors. This process takes the doctors too much time and energy. Besides, doctors often work for long time, their judgments are also likely to be negatively affected by their body fatigue. To solve the problem, automatic detection technology of epilepsy EEG is born.

At present, there are abundant researches of automatic detection based on epilepsy EEG. However, only a few literatures have focused on the analysis of seizure prediction. In real life, it is meaningful to predict seizures. For patients, uncertainty of seizures may cause unpredictable accidents, which may seriously affect life and work (Holmes, 1984; Ahmed, 2005). The effective prediction of seizure can help patients to solve the problems in time, thus reducing the loss of patients to a minimum. In addition, for doctors and researchers, the effective prediction of seizures not only helps them to explore the basic mechanisms of epileptic seizures, but also provides important support for building accurate and stable epilepsy auxiliary diagnostic tools. In this paper, we will focus on seizure prediction.

The process of seizure prediction generally consists of EEG signal acquisition, data preprocessing, feature extraction, and classification. In particular, mining rich and effective features from native EEG data is essential to improve classification accuracy. According to variation of the domains that EEG features are extracted from, features may come from time domain, frequency domain, and the time-frequency domain.

In time domain, Tessy et al. (2016) focused on extracting two time-domain features of line length and energy to obtain high classification results on k-nearest neighbors (KNN) classifier. The algorithm proposed by Shanir et al. (2015) was based on average and minimum of each segment. In addition, the team of Zhang et al. (2018) divided EEG into several clusters and extracted a set of time-domain features from each cluster. Each group of features was regarded as a node of complex network. Then, average weighted degree was calculated from network as classification feature.

In frequency-domain, through experiments on Freiburg and CHB-MIT databases, Zhou et al. (2018) proved that classification accuracy of frequency-domain signals is significantly better than that of time-domain signals. Al Ghayab et al. (2019) extracted statistical features from sub windows by Fourier transform, and sorted features by using information gain technique to select the most appropriate ones.

In time-frequency-domain, more and more researches focused on extracting EEG features from time-frequency distribution (TFDs). Guerrero-Mosquera et al. (2010) extracted length, frequency, and energy from the smoothed Wigner-Ville distribution (SWVD) by using trajectory estimated from McAulay-Quatieri sinusoidal model. A new method (Wavelet-Chaos) of wavelet transformation was proposed by analyzing δ, θ, α, and β subbands of EEG, they found that significant differences could be captured by combining subband information (Adeli et al., 2007). Sharma et al. (2014) used cubical threshold denoising methods based on wavelet to analyze EEG signals before extracting statistical features from frequency bands (0 ~ 8, 8 ~ 16, 16 ~ 32, and 0 ~ 32 Hz). In addition, local binary pattern (LBP) was extended to analysis of EEG signals, because of its outstanding advantages such as rotation invariance and gray invariance. For example, Shanir et al. (2018) proposed a morphological feature extraction method based on LBP operator.

Considering comprehensively above these points, we propose multi-dimensional enhanced seizure prediction framework based on graph convolutional network (MESPF). The contributions of this research are as follows:

- It is very importance to improve accuracy of seizure prediction. To provide the prediction model with more powerful and abundant data, we enhance overall consideration of dimensions about epilepsy EEG signals. In particular, we take multichannel spatial relationship as breakthrough point, update representation of graph relational data at frequency band level, and explore the space-time relationships.
- We propose a multi-dimensional enhanced seizure prediction framework, which mainly includes information reconstruction space, graph state encoder, and space-time predictor. We combine technical advantages of wavelet packet decomposition, graph convolutional network and gated recurrent neural network in construction of the framework. From aspects of frequency, channel, and time, we intend to mine richer and more effective data information than previous studies.
- Finally, we apply the framework to data set (CHB-MIT) for verification. In terms of experimental results, it has surpassed or approached many existing algorithms. In short, our framework provides a novel way for peers to study principle of seizure prediction.

The organizational structure of our article is as follows, the second section introduces prediction principle of the framework (MESPF), the third section briefly describes experimental results and analysis, and the fourth section is summary.

## 2. Methodologies

**Figure 1:**
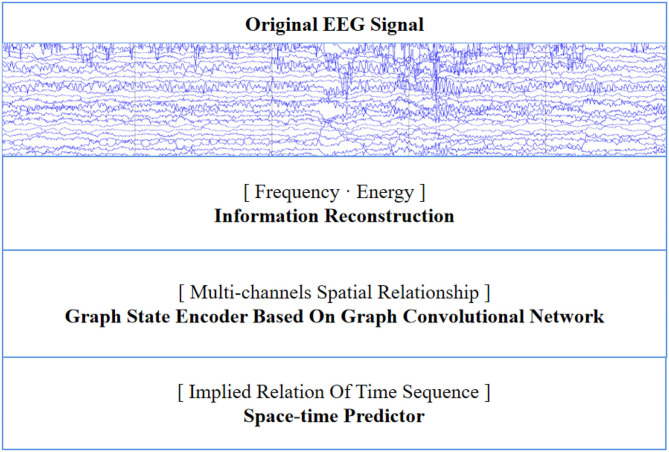
Our model mainly includes information reconstruction space, graph state encoder, and space-time predictor.

For seizure prediction, we propose a multi-dimensional enhanced seizure prediction framework (MESPF). It mainly includes information reconstruction space, graph state encoder, and space-time predictor. The main process of the model is shown in Figure 1. The following contents give specific explanations in turn.

### 2.1. Information Reconstruction Space

Patients vary in physiological mechanisms, and their pre-ictal signals may occur in different frequency band range. To capture these subtle difference, we designs an information reconstruction space. It is mainly used to highlight specificity and explore the effect of feature enhancement on graph encoding.

Since epileptic EEG signals are random, non-stationary and non-linear, we actively introduce wavelet packet decomposition (WPD) in information reconstruction space to decompose EEG signals. It should be emphasized that since range of wavelet transform is mainly low frequency part of signals, it is difficult to characterize a large amount of detailed information. However, wavelet packet decomposition can orthogonally decompose signal in full frequency range, and resolution of high frequency part is better than the former. It is a more precise analysis method than wavelet transform. So that it has gradually become one of main methods for analyzing non-stationary signals (Hyvarinen et al., 2001). Through wavelet packet decomposition, we can analyze EEG signals from multiple frequency bands.

**Figure 2:**
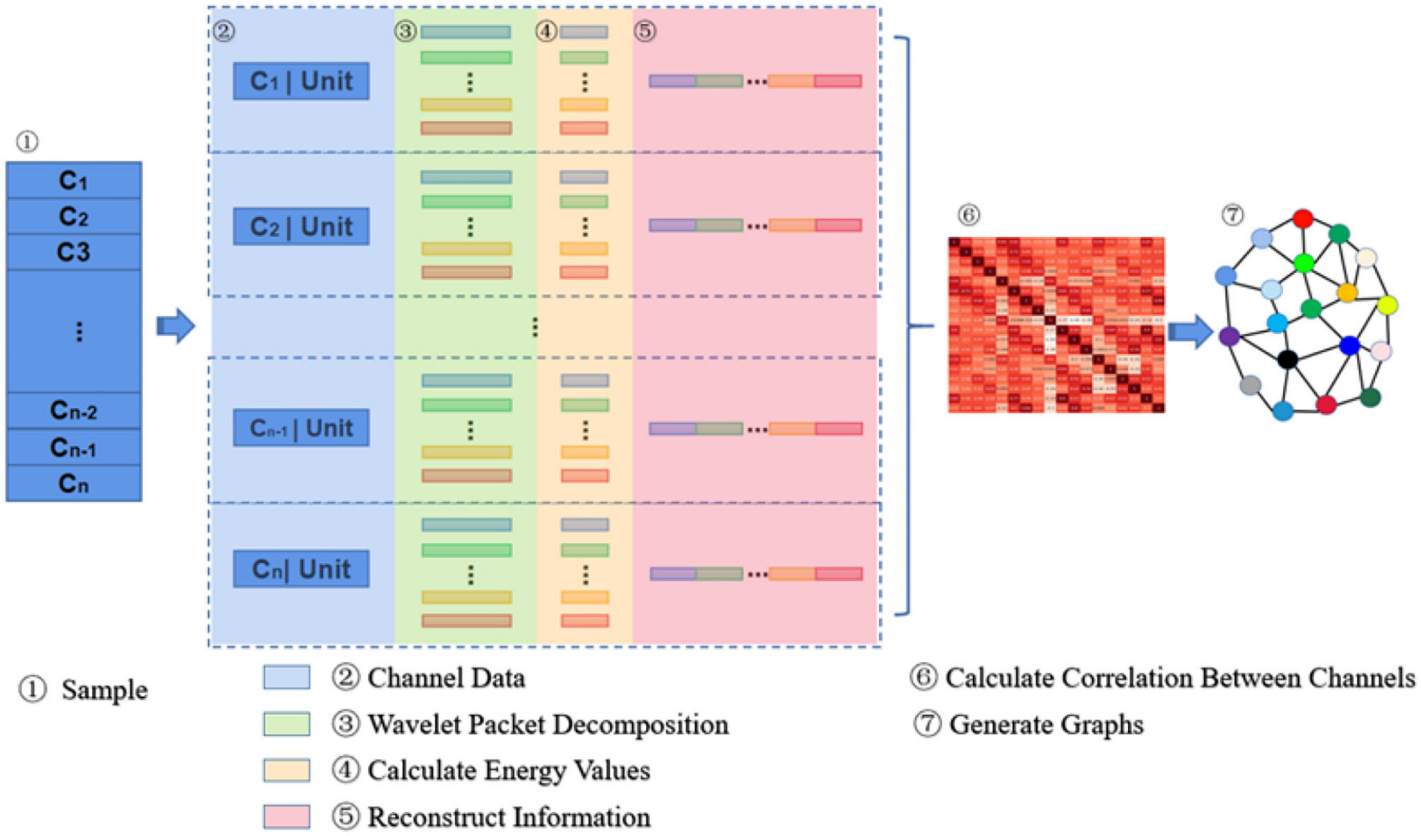
The figure shows concrete principle of information reconstruction space.

The principle of information reconstruction space in MESPF is shown in Figure 2. By decomposing epileptic EEG signals and calculating characteristics of energy value, it can update representation of graph data. First of all, *C*_1_ − *C*_*n*_ represents 18 channels. In the third step, data of each channel is processed by wavelet packet decomposition technique. Then we calculate energy values of corresponding sub-bands and reconstruct vectors characterized by energy values (step 4–5). After completion of channel data reconstruction, we use Pearson correlation coefficient calculation method to calculate correlation between multiple channels (step 6). We update graph relationship representation as one of direct inputs to graph encoder (equivalent to edges of graph). Finally, related relational data and each channel data itself (equivalent to vertex data of graph) are used as input to graph encoder, as shown in step 7.

**Figure 3:**
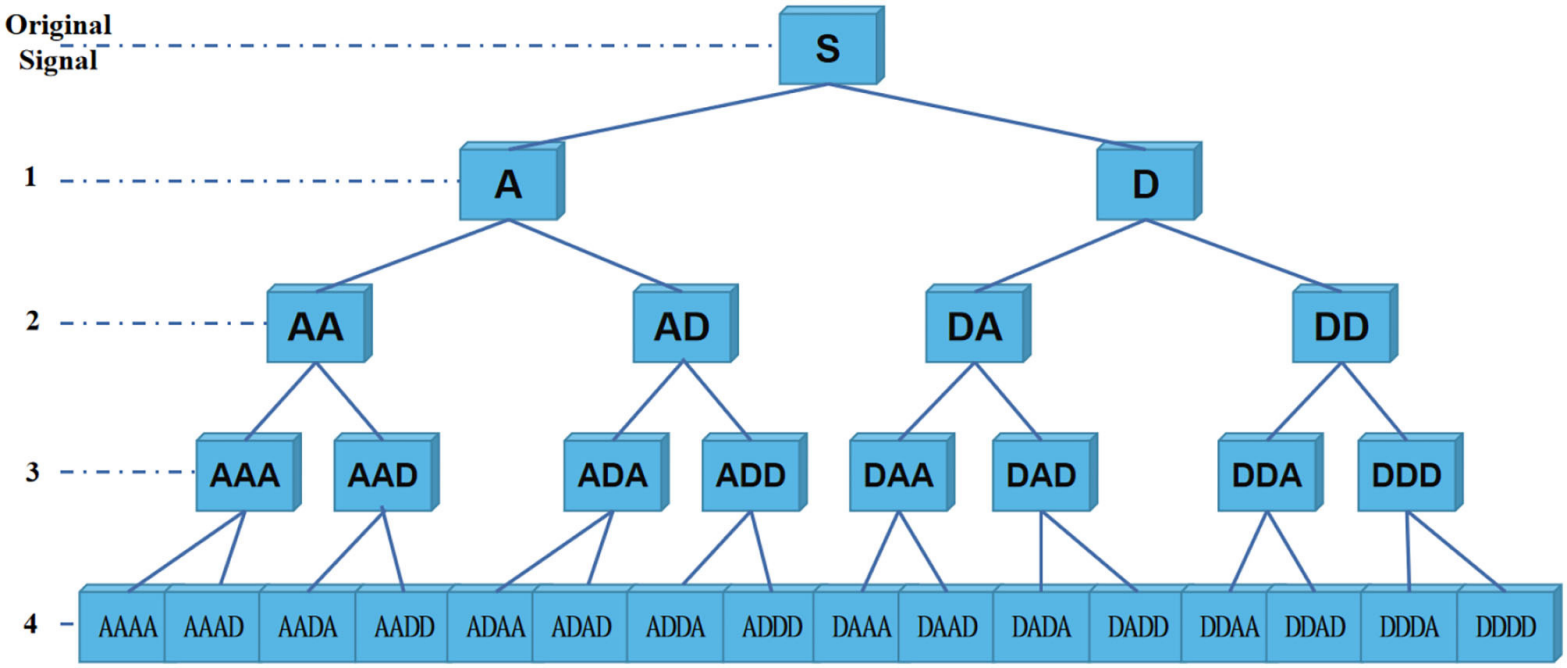
EEG signals are divided into several sub-bands by wavelet packet decomposition.

Specifically, decomposition principle of information reconstruction space is shown in Figure 3. For a signal, wavelet coefficients (A and D) are obtained by wavelet packet decomposition of the first layer. A represents low frequency part of signal, and D represents high frequency part. Each node in graph represents a data sequence. Then, energy values of each sub-band are calculated, respectively. In the experiment, four-layer wavelet packet decomposition is adopted to obtain wavelet coefficients of 16 frequency bands. In this way, each signal unit can be represented by energy values. It should be noted that since epilepsy EEG signal is continuous waveform, our experiment uses a relatively smooth Daubechies (Db) wavelet base.

### 2.2. Graph State Encoder

After signal unit is processed by information reconstruction space, the graph data are given into graph encoder for further feature processing.

**Figure 4:**
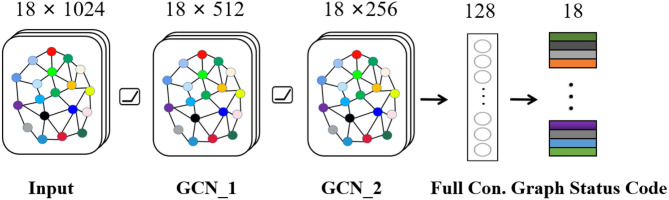
Graph state encoder mainly includes input layer, graph convolution layer, and fully connected layer.

Seizure is a synergistic result of multiple brain regions. For EEG, each channel records activities of different brain regions, and there must be a certain relationship between channels. Therefore, we build a graph state encoder to explore relationship by extracting spatial features between multiple channels. The specific structure and parameters about graph encoder are shown in Figure 4, which mainly includes input layer, graph convolution layer-1, graph convolution layer-2, fully connected layer, and graph status code layer. The activation layer in network uses Rectified Linear Unit (ReLU), which can speed up convergence speed. In general, after extracting features of graph space through graph convolution layer, relevant data are weighted by fully connected layer. Then, graph status code is generated. As for status code, it is composed of 18 eigenvalues, which, respectively, represent 18 channels of graph data. Each status code represents data characteristics of corresponding time period. Finally, we feed status codes into space-time predictor in chronological order.

As for graph state encoder, we refer to method proposed by Kipf and Welling (2016) and build sub-module based on graph convolutional network. In particular, we need to emphasize application of filter. Firstly, we define convolution operation on graph as:

(1) $$\begin{array}{r} \begin{array}{c} Conv=X_{MESPF}*K_{e} \end{array} \end{array}$$

*X*_*MESPF*_ represents direct input (signal) of graph state encoder, *K*_*e*_ represents convolution kernel. Furthermore, in previous studies, Defferrard et al. (2016) introduced Chebyshev polynomials, and they provided an algorithm for constructing fast local filters in spectral domain, which can learn local, static and combined features on graph. Subsequently, Kipf and Welling (2016) optimized convolutional network structure through the first-order local approximation of spectrogram convolution. In process of graph convolution, due to existence of graph Fourier transform, computational complexity of model is relatively high. Chebyshev polynomials have numerical stability and computational efficiency in field of polynomial function approximation. Therefore, we actively introduce Chebyshev polynomials into the model. The relevant formula is as follows:

(2) $$\begin{array}{r} \begin{array}{c} T_{k}\left(X_{MESPF}\right)=2X_{MESPF}T_{k-1}\left(X_{MESPF}\right)-T_{k-2}\left(X_{MESPF}\right) \end{array} \end{array}$$

(3) $$\begin{array}{r} \begin{array}{c} K_{e}\left(\Lambda \right)\approx \sum_{n=0}^{N}\theta_{n}T_{n}\left(\tilde{\Lambda }\right) \end{array} \end{array}$$

θ represents vector of Chebyshev coefficients, $\tilde{\Lambda }=\frac{2}{\lambda_{max}}\Lambda -I$, Λ represents diagonal matrix of eigenvalues, λ_*max*_ represents maximum eigenvalue of regularized feature matrix, *I* represents identity matrix, convolution on graph is finally expressed as:

(4) $$\begin{array}{r} \begin{array}{c} O_{MESPF}=X_{MESPF}*K_{e}\approx \sum_{n=0}^{N}\theta_{n}T_{n}\left(\tilde{\Lambda }\right)X_{MESPF} \end{array} \end{array}$$

*O*_*MESPF*_ represents the output of graph convolution layer. Then *O*_*MESPF*_ is processed through ReLU activation function, this is shown in Formula 5.

(5) $$\begin{array}{r} \begin{array}{c} O_{MESPF}^{*}=ReLU\left(O_{MESPF}\right)=max\left(0,O_{MESPF}\right) \end{array} \end{array}$$

Finally, through fully connected layer, we synthesize several graph space features to generate status code *S*_*MESPF*_.

(6) $$\begin{array}{r} \begin{array}{c} S_{MESPF}=FC{\left(O_{MESPF}^{*}\right)}_{18} \end{array} \end{array}$$

### 2.3. Space-Time Predictor

In the model, space-time predictor is constructed. After exploring multi-channel spatial relationship, we further explore change rule at time series level.

**Figure 5:**
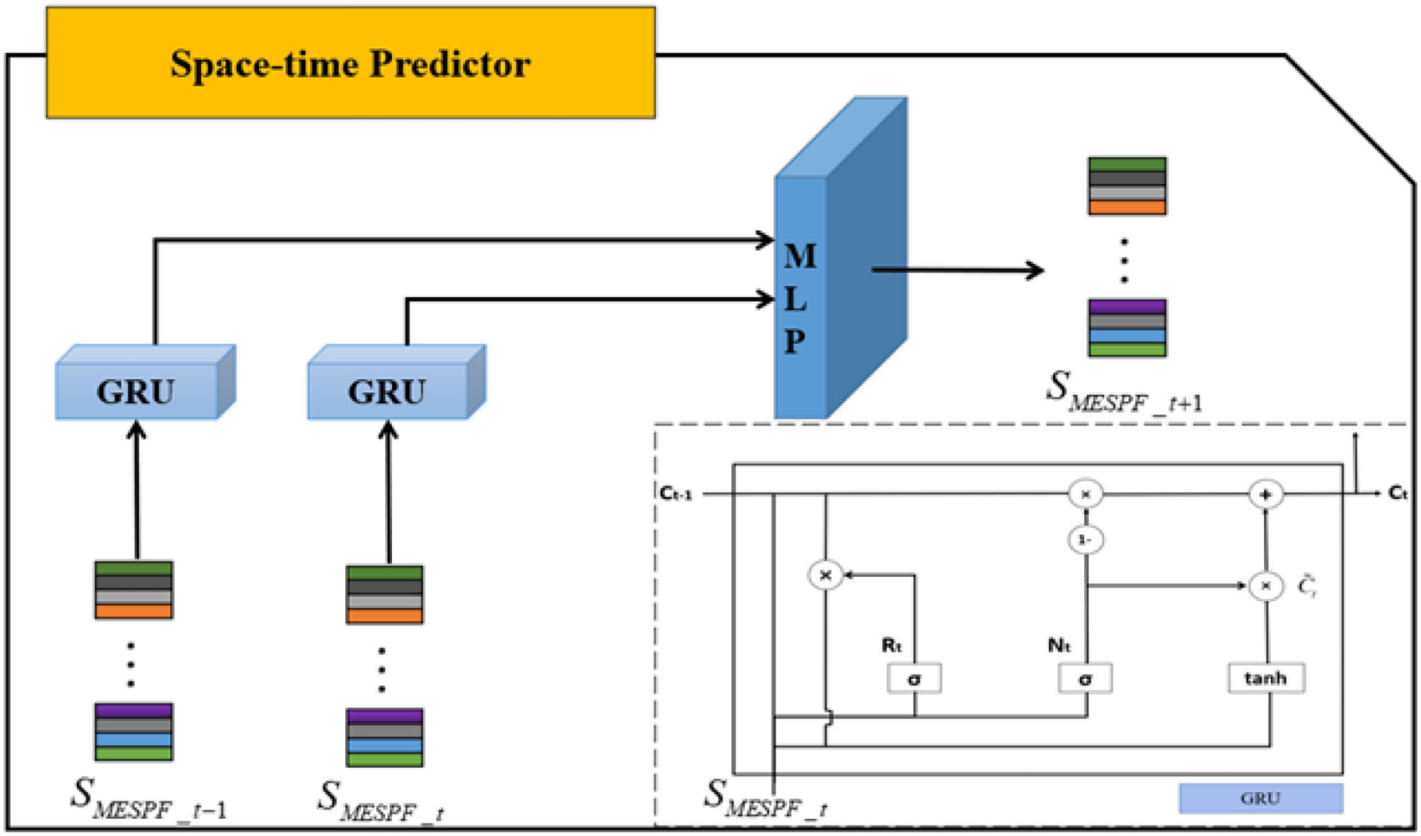
The core part of space-time predictor is gate recurrent unit.

The internal structure of space-time predictor is shown in Figure 5. *S*_*MESPF*_*t*−1_ and *S*_*MESPF*_*t*_, respectively, represent graph status codes at time *t* − 1 and time *t*. Specifically, as complexity of neural network architecture increases or training time of experimental data becomes longer, phenomenon of gradient disappearance or explosion is easy to occur. It is difficult to master law of EEG timing signal. The emergence of gate recurrent network solves related problems well. So, it is mainly built on the basis of gate recurrent unit. And direct input of space-time predictor is output of graph state encoder. It should be emphasized that relevant input data should be entered in chronological order. By mining implicit relationship in terms of timing, prediction results are finally output by multilayer perceptron (MLP).

**Figure 6:**
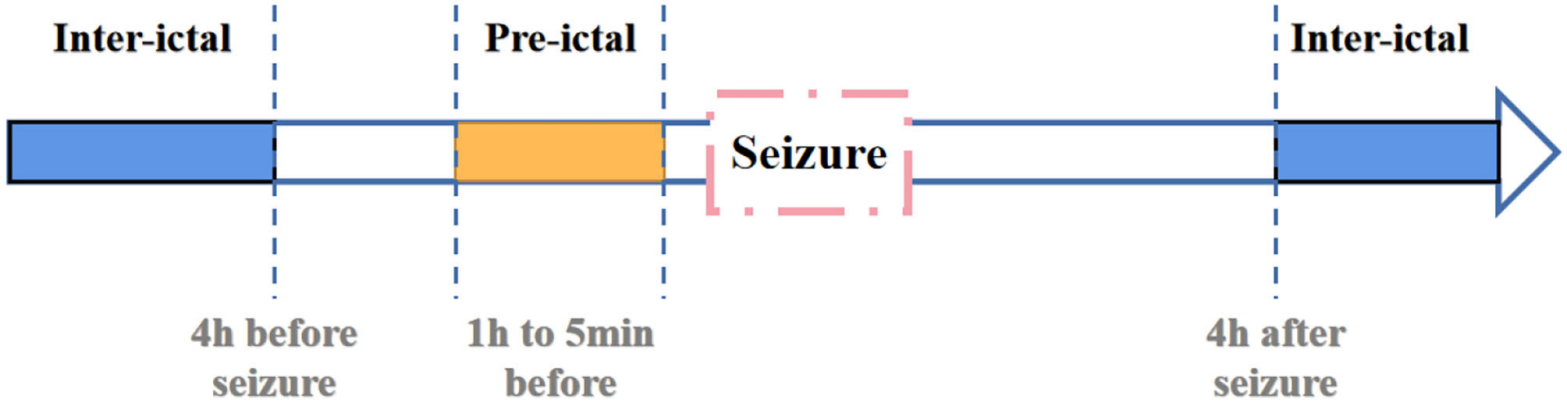
According to actual seizure process, EEG is divided into different stages.

Focus on gated recurrent unit, Cho et al. (2014) integrate forget gate and input gate into an update gate. Specifically, GRU includes update gate and reset gate. The former controls extent to which state information at previous moment is brought into the current state. And the latter controls extent to which state information at previous moment is ignored. The GRU structure is shown in lower right corner of Figure 6. Then, we combine diagram and formulas to further explain.

The calculations for reset gate (*R*_*t*_) is shown in Formula (5). Both *W*^*r*^ and *U*^*r*^ are weight matrices. And σ represents sigmoid function, which can map results between 0 and 1. The calculation principle of update gate (*N*_*t*_) is similar to that of reset gate.

(7) $$\begin{array}{r} \begin{array}{c} R_{t}=\sigma \left(W^{r}S_{MESPF\text{\_}t}+U^{r}C_{t-1}\right) \end{array} \end{array}$$

(8) $$\begin{array}{r} \begin{array}{c} N_{t}=\sigma \left(W^{z}S_{MESPF\text{\_}t}+U^{z}C_{t-1}\right) \end{array} \end{array}$$

The calculation of candidate hidden layer (${\tilde{C}}_{t}$) is as follows. Tanh represents tanh function, and its mapping range is −1 to +1.

(9) $$\begin{array}{r} \begin{array}{c} {\tilde{C}}_{t}=\tanh\left(WS_{MESPF\text{\_}t}+R_{t}UC_{t-1}\right) \end{array} \end{array}$$

The final output hidden layer information (*C*_*t*_) calculation formula is as follows:

(10) $$\begin{array}{r} \begin{array}{c} C_{t}=\left(1-N_{t}\right)*C_{t-1}+N_{t}*{\tilde{C}}_{t} \end{array} \end{array}$$

## 3. Results Analysis

### 3.1. Data Set Description

In order to prove effectiveness of the framework, we further apply it to CHB-MIT data set. The download link for complete database is https://physionet.org/content/chbmit/1.0.0/. It consist of 23 data samples from 22 subjects(5 males, ages 3–22; and 17 females, ages 1.5–19). All samples are stored in EDF format. And all signals are sampled at 256 samples per second. It should be noted that this is a verification experiment of validity. In order to achieve effective prediction of seizures, we uses the framework to classify pre-ictal and inter-ictal.

In previous studies, Litt et al. (2001) have demonstrated that seizure-like EEG signals become more frequent at 2 h before actual seizure. They propose that accumulated energy will increase within 50 min before initial state. And Affes et al. (2019) propose that pre-seizure phase is 1 h before seizure. Based on researches mentioned above, we defines pre-ictal period as data between 1 h and 5 min before seizure. And the definition of inter-ictal is shown in Figure 6.

It should be noted that in the process of data collection, data of inter-ictal period is much more than data of pre-ictal period. To make the number of samples in these two periods equal, an overlapped window is applied in pre-ictal period for data segmentation, and the window overlap rate is set as 50%.

### 3.2. Experimental Indicators

The evaluation indicators of our model include Accuracy, Sensitivity, Specificity, False Positive Rate, and F1-Score. The calculation formulas for these indicators are as follows:

(11) $$\begin{array}{r} \begin{array}{c} Accuracy=\frac{TP+TN}{TP+TN+FP+FN}\times 100 \end{array} \end{array}$$

(12) $$\begin{array}{r} \begin{array}{c} Specificity=\frac{TN}{TN+FP}\times 100 \end{array} \end{array}$$

(13) $$\begin{array}{r} \begin{array}{c} Sensitivity=\frac{TP}{TP+FN}\times 100 \end{array} \end{array}$$

(14) $$\begin{array}{r} \begin{array}{c} False\;Positive\;Rate=\frac{FP}{FP+TN}\times 100 \end{array} \end{array}$$

(15) $$\begin{array}{r} \begin{array}{c} Positive\;Predictive\;Value\left(PPV\right)=\frac{TP}{TP+FP}\times 100 \end{array} \end{array}$$

(16) $$\begin{array}{r} \begin{array}{c} Negative\;Predictive\;Value\left(NPV\right)=\frac{TN}{TN+FN}\times 100 \end{array} \end{array}$$

(17) $$\begin{array}{r} \begin{array}{c} F1-Score=\frac{2TP}{2TP+FP+FN} \end{array} \end{array}$$

TP indicates that it is actually a positive example and the predicted result is a positive example. FP is actually a negative example, and the predicted result is a positive example. TN indicates that it is actually a negative example, and predicted result is a negative example. FN indicates that it is actually a positive example, and predicted result is a negative example.

### 3.3. Experimental Environment Configuration

As for experimental environment, information reconstruction space of MESPF model is completed on the Windows10 system with Intel(R) Core(TM) I7-6500U CPU @ 2.50GHz 2.59GHz. Construction of graph encoder and space-time predictor is completed on the Ubuntu system. Relevant model is built based on TensorFlow framework with Python. Adam optimizer is also used in related experiments, and initial learning rate is set at 0.01.

In addition, the loss function of the model includes cross entropy term and L2 regularization term, as shown in Formula (18). *H*(*p*_*MESPF*_*O*_,*q*_*MESPF*_*R*_) represents the loss function of MESPF model for seizure prediction. *p*_*MESPF*_*O*_ represents related predicted value of space-time predictor. *q*_*MESPF*_*R*_ represents true label for each data unit.

(18) $$\begin{array}{l} H(p_{MESPF\_O,}q_{MESPF\_R})=\sum_{x}P_{MESPF\_O}(x)\cdot \log(\frac{1}{q_{MESPF\_R}(x)})\\ \;+\frac{\lambda }{2}\sum w^{2} \end{array}$$

### 3.4. Analysis of Experimental Results

**Figure 7:**
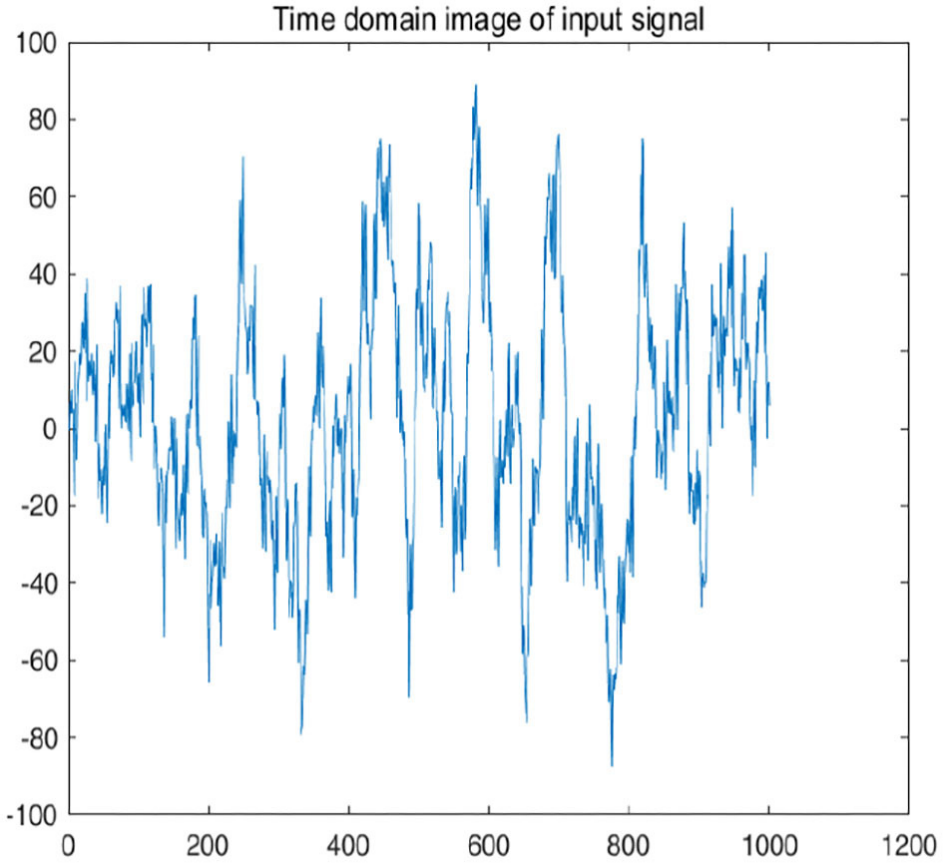
Time-domain image of signal in pre-ictal is shown in figure.

**Figure 8:**
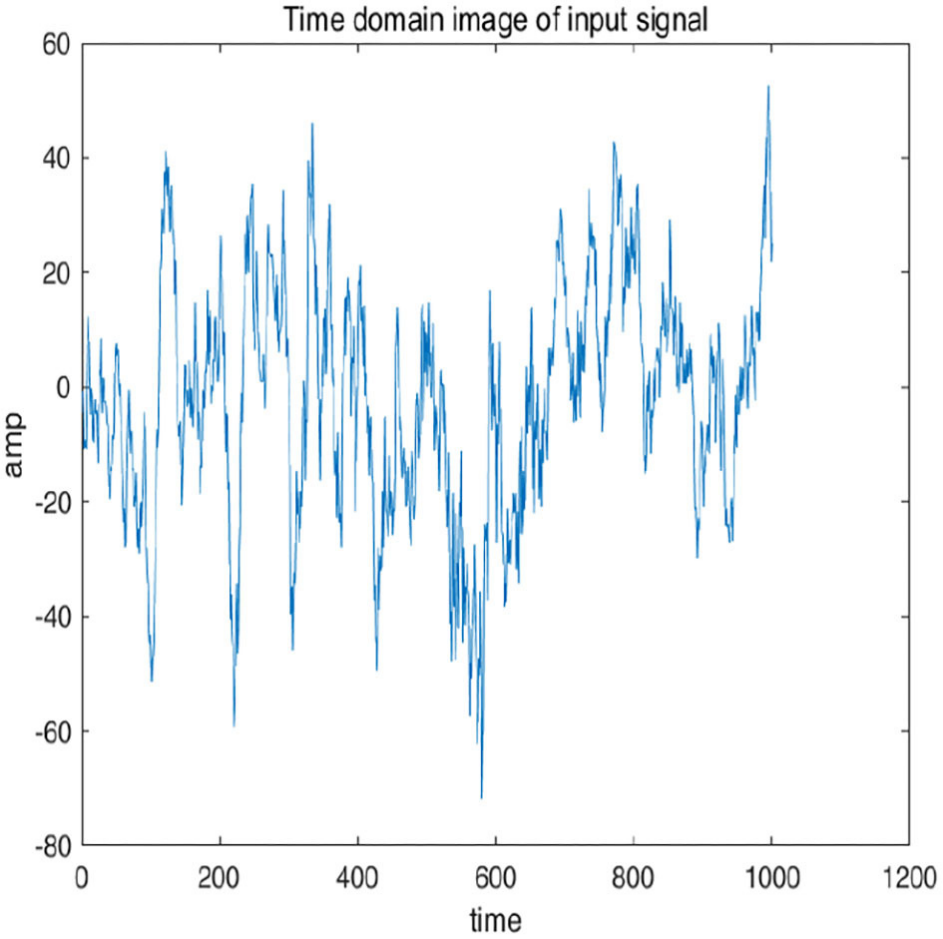
Time-domain image of signal in pre-ictal is shown in figure.

**Figure 9:**
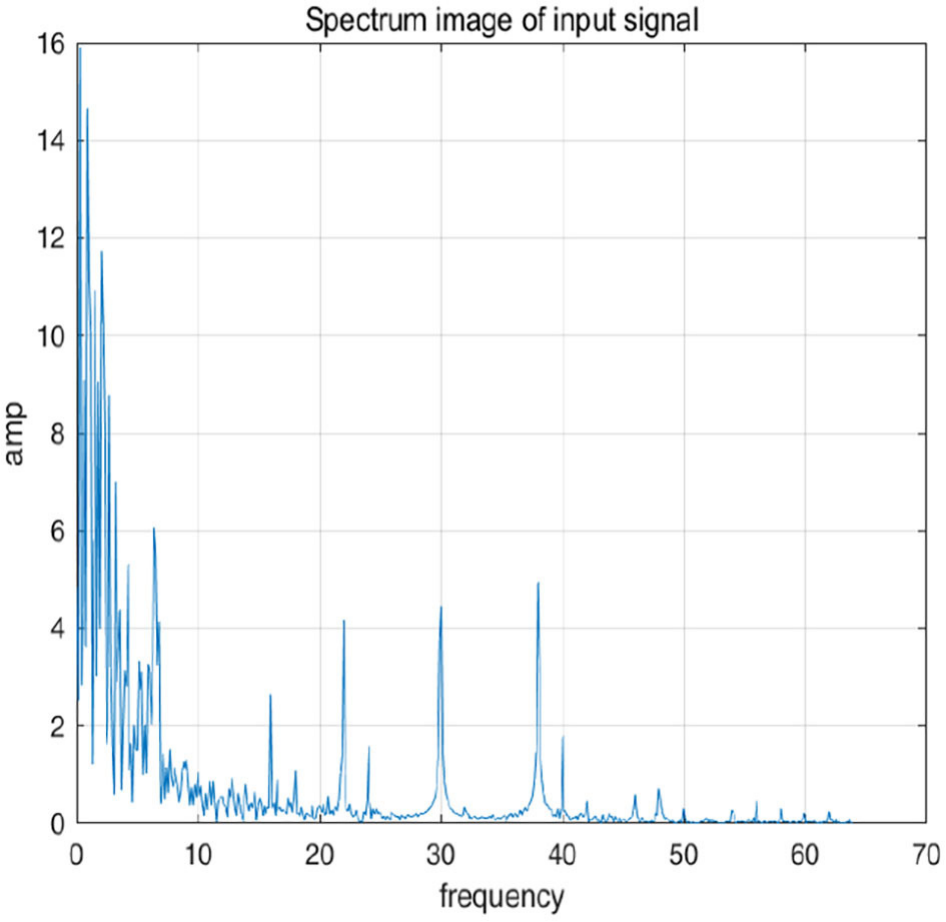
Frequency-domain image of signal in inter-ictal is shown in figure.

**Figure 10:**
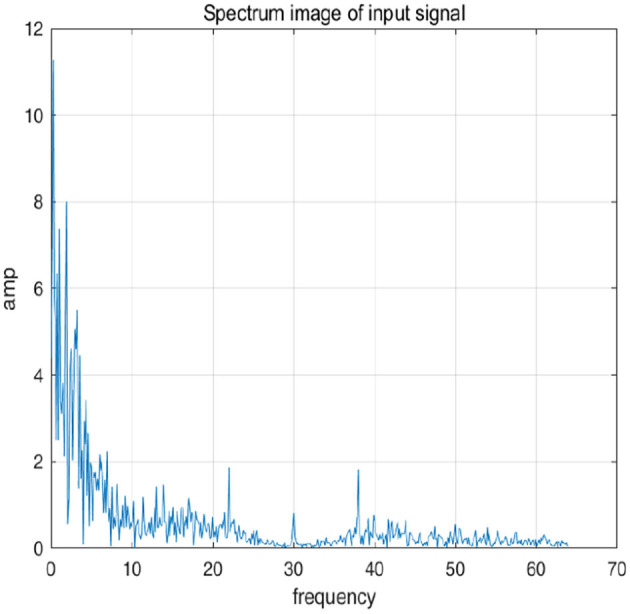
Frequency-domain image of signal in inter-ictal is shown in figure.

To further explain principle of information reconstruction space, we set up a control group to visualize the process of reconstruction. One group is pre-ictal samples, and the other group is inter-ictal samples. Figures 7, 8 show time domain information. It can be seen that there are more high frequency signals before seizure. These are extremely important features for predicting seizure in advance. Figures 9, 10 are images after transforming from time domain to frequency domain. It can also be seen from the former that times of high frequency increases significantly in pre-ictal period.

**Figure 11:**
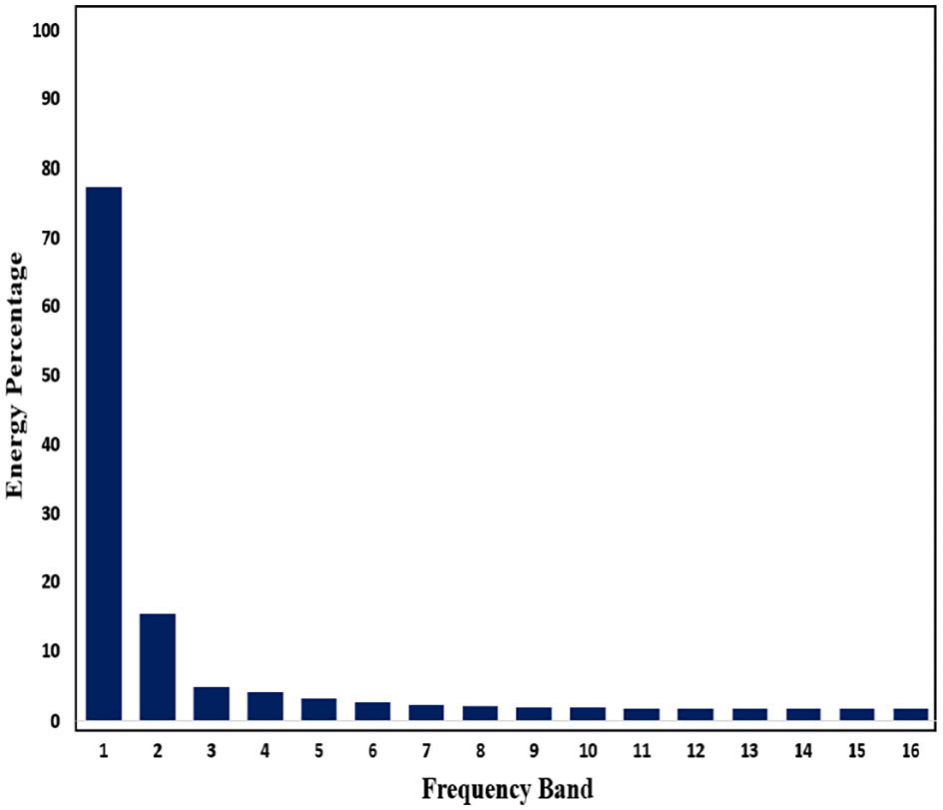
Energy percentage of each frequency band (pre-ictal) in the fourth layer of WPD is shown in figure.

**Figure 12:**
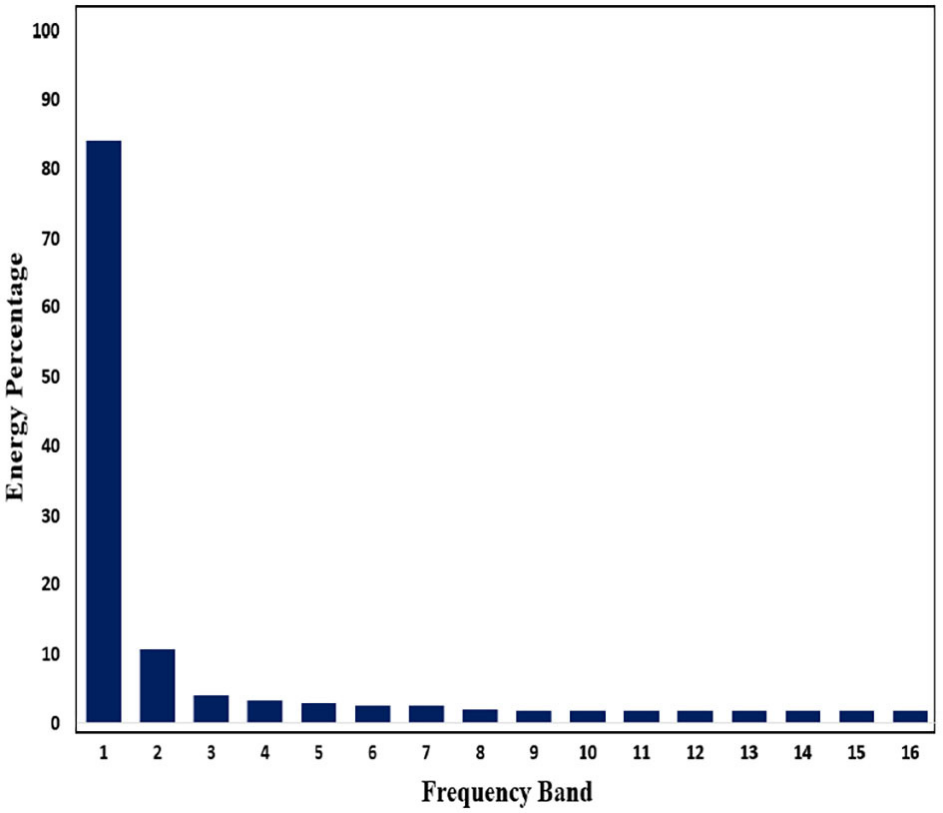
Energy percentage of each frequency band (inter-ictal) in the fourth layer of WPD is shown in figure.

Subsequently, in order to mine the law of different frequency band in signals, we use WPD to decompose signals. Through WPD, we extract wavelet packet coefficients of nodes. Then energy of wavelet packet coefficients is used as eigenvalue to construct eigenvector. Figures 11, 12 show energy values of the fourth layer after four-layer WPD.

In our experiment, each epileptic patient is assigned a unique ID number, from case 1 to case 24. The comprehensive index analysis for each patient is shown in Table 1. These indicators include specificity, sensitivity, PPV, NPV, FPR, and accuracy. More than half of the experiments achieve sensitivity of 100%. The average level of false positive rate is only 0.0106. Experimental data shows that the lower false positive rate, the better model's performance. For details of other experimental indicators, please refer to Table 1.

**Table 1:** Specific indicators of samples are shown in the table.

**ID**	**Specificity**	**Sensitivity**	**PPV**	**NPV**	**FPR**	**Accuracy**
1	98.85	100.00	98.87	100.00	0.0115	99.43
2	100.00	100.00	100.00	100.00	0.0000	100.00
3	99.67	100.00	99.67	100.00	0.0033	99.84
4	97.82	97.85	97.81	97.85	0.0219	97.83
5	100.00	100.00	100.00	100.00	0.0000	100.00
6	92.85	95.38	93.02	95.26	0.0715	94.11
7	98.65	100.00	98.66	100.00	0.0135	99.32
8	100.00	99.34	100.00	99.34	0.0000	99.67
9	100.00	100.00	100.00	100.00	0.0000	100.00
10	100.00	100.00	100.00	100.00	0.0000	100.00
11	99.24	94.56	99.20	94.80	0.0076	96.90
12	98.46	94.50	98.39	94.71	0.0155	96.48
13	96.98	96.24	96.96	96.27	0.0302	96.61
14	99.02	100.00	99.04	100.00	0.0097	99.51
15	98.63	97.05	98.61	97.10	0.0137	97.84
16	97.76	100.00	97.81	100.00	0.0224	98.88
17	99.12	100.00	99.12	100.00	0.0089	99.56
18	99.42	97.04	99.41	97.11	0.0057	98.23
19	100.00	100.00	100.00	100.00	0.0000	100.00
20	100.00	97.18	100.00	97.26	0.0000	98.59
21	100.00	100.00	100.00	100.00	0.0000	100.00
22	100.00	100.00	100.00	100.00	0.0000	100.00
23	98.37	98.31	98.37	98.31	0.0163	98.34
24	99.85	99.26	99.84	99.26	0.0016	99.55
Average	98.95	98.61	98.95	98.64	0.0106	98.78

**Figure 13:**
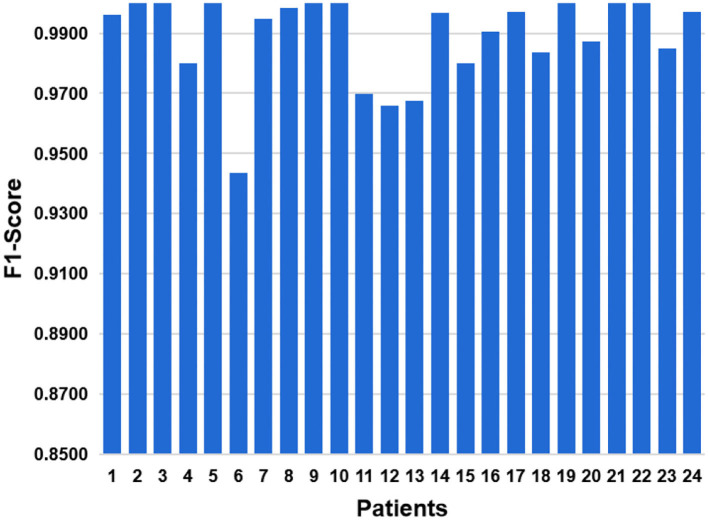
In order to balance influences of precision and recall, we have added F1-Score indicator.

As a comprehensive index, F1-Score balances effects of precision and recall, and can systematically evaluate a classifier. The value of F1-Score ranges from 0 to 100%, and the larger the F1 value, the better the model performance. The result of F1-Score is shown in Figure 13. The average level of 24 cases is 0.9877. The best F1-Score value reach 100% for patients with ID 2, 3, and 5. And the lowest F1-score is 0.9419 for patient with ID 6.

Finally, we compare the performance of the proposed model with some existing algorithms, from traditional ones to that based on deep learning. The results are shown in Table 2. In terms of our method, we have analyzed epileptic EEG signals from multiple dimensions, including energy characteristics of frequency bands, spatial characteristics of channels, and timing characteristics of signals. In the process, we make full use of advantages of wavelet packet decomposition, graph convolutional network and gate recurrent unit network in their professional fields.

**Table 2:** In classification tasks of pre-ictal and inter-ictal, the framework is compared with other advanced methods.

**References**	**Years**	**Method**	**Specificity**	**Sensitivity**	**Accuracy**
Khan et al. (2012)	2012	LDA	100.00	83.00	91.80
Kiranyaz et al. (2014)	2014	Automated patient-specific	94.71	89.00	-
Pramod et al. (2014)	2015	NN	99.29	98.06	-
Alotaiby et al. (2015)	2015	CSP	98.61	86.84	92.72
Yuan et al. (2018)	2018	Multi-view DL	-	-	94.37
Solaija et al. (2018)	2018	Dynamic mode decomposition	98.93	87.00	-
Dash et al. (2020)	2019	IFD and HMM	99.85	96.78	99.60
Our method	2020	MESPF	98.95	98.61	98.78

As we all know, sensitivity is the proportion of people who are actually sick that are correctly judged as true positives. It is a meaningful indicator in medical clinical diagnosis. Table 2 shows an excellent performance of our model in sensitivity (98.61%). Besides, the accuracy is also consistent with the high level of other algorithms, and the specificity also reaches the average level of the other algorithms.

## 4. Conclusions

In conclusion, we propose the MESPF framework to explore the law of epilepsy EEG signals. From frequency, channel and time, we, respectively, build information reconstruction space based on wavelet packet decomposition, graph state encoder based on graph convolution network, and space-time predictor based on gated recurrent unit. We make full use of advantages of different methods to build an efficient seizure prediction framework (MESPF). MESPF has achieved better results in classification of pre-ictal and inter-ictal than existing methods. This integrated method of multidimensional and multi-method epileptic EEG provides a more novel idea for peers to study biomedical signals.

As far as the research is concerned, we take frequency, space (channel), and time into consideration. However, there is still a lot of work to be done. In our next step, we will consider more features, such as multiple spikes, approximate entropy, information entropy, fuzzy entropy, etc. Finally, we hope to build a more accurate and stable intelligent framework for seizure prediction by continuously mining surface meaning and internal correlation of epileptic EEG.

## Data Availability Statement

Publicly available datasets were analyzed in this study. This data can be found here: https://www.physionet.org/content/chbmit/1.0.0/.

## Author Contributions

XC: conceptualization, methodology, software, and writing—reviewing and editing. YZ: conceptualization, methodology, supervision, and funding acquisition. CD: validation, formal analysis, and data curation. SS: conceptualization, methodology, and writing—reviewing and editing. All authors contributed to the article and approved the submitted version.

## Conflict of Interest

The authors declare that the research was conducted in the absence of any commercial or financial relationships that could be construed as a potential conflict of interest.

## Publisher's Note

All claims expressed in this article are solely those of the authors and do not necessarily represent those of their affiliated organizations, or those of the publisher, the editors and the reviewers. Any product that may be evaluated in this article, or claim that may be made by its manufacturer, is not guaranteed or endorsed by the publisher.
